# Mechanism of strength deterioration of red sandstone on reservoir bank slopes under the action of dry–wet cycles

**DOI:** 10.1038/s41598-023-47397-x

**Published:** 2023-11-16

**Authors:** Chao Chen, Baoyun Zhao, Liyun Zhang, Wei Huang

**Affiliations:** 1 CCTEG Chongqing Engineering (Group) Co., LTD, Chongqing, 400016 China; 2 School of Civil Engineering, Chongqing University, Chongqing, 400045 China; 3 School of Civil Engineering and Architecture, Chongqing University of Science and Technology, Chongqing, 401331 China; 4 Finance Bureau of Korla City, Bazhou, 841000 Xinjiang China

**Keywords:** Civil engineering, Petrology

## Abstract

To investigate the micro-scale mechanism of strength deterioration under different times of dry–wet cycles, laboratory tests of physical properties, triaxial compression, X-ray diffraction (XRD) and scanning electron microscopy (SEM) were conducted on red sandstone on reservoir bank slopes. The research results showed that when the 5th dry–wet cycle ended, the dry mass and peak strength started to rapidly decline, while the porosity and saturated mass increased rapidly. In general, all of these behaviours become relatively stable when the number of cycles increased. Similarly, cohesion and internal friction angle changed most significantly from 0 to 10 cycles and then became stable. In addition, the physical expansion of the adsorbed water film and the dissolution and expansion of mineral particles increased the number of internal microcracks and pores and the porosity and saturated mass of the sample. In addition, the pore fluid effect and fracture flow effect made the microcracks in the red sandstone grow and connect; thus, the peak strength of the sample decreased. Moreover, during the dry–wet cycles, the change in the relative content of mineral particles and the pore fluid effect weakened the clay cementation, and then the dry mass and cohesion of the samples decreased. The research reported in this paper will play a very significant role in the scientific analysis of slope stability in the Three Gorges Reservoir area.

## Introduction

The red sandstone sediments flanking the Yangtze River within the Three Gorges Reservoir region represent an assortment of inland lacustrine sedimentary deposits characterized by a notable abundance of argillaceous clay minerals. These sedimentary formations undergo a distinct pattern of disintegration and transformation into mud deposits, primarily attributed to the cyclic fluctuations in moisture levels that correspond to the oscillations in reservoir water levels, manifesting as periods of high and low water levels^1,2^. The intricate mechanisms governing the deterioration of rock and soil within this context arise from the interplay of various factors. Notably, alterations in the microstructural composition of rocks upon contact with water emerge as the predominant contributors to the diminishment of their mechanical strength^3,4^.

Over the past decade, extensive experimentation by scholars has focused on investigating how water influences the weakening of various rock types under varying conditions. Colback et al.^5^ conducted comparative studies involving different water contents, revealing that the strength of rocks in a saturated state is merely half that of their dry state counterparts. This unequivocally establishes water as the primary factor behind rock strength degradation. Wasantha et al.^6^ conducted triaxial compression tests, demonstrating that water’s detrimental effect on rocks persists under varying in-situ stress conditions. Given the fluctuating nature of water levels in natural environments, it becomes imperative to scrutinize strength degradation in response to dry–wet cycles. Fu et al.^7^ observed that within the dry–wet cycle, the initial stages exhibited more pronounced deterioration, while the later stages saw a decline in this effect. Similar findings were reported by Liu et al.^8^, where the decrease in strength and increase in strain diminished with an increasing number of dry–wet cycles. In order to further explore the role of wet-dry cycles in strength deterioration. Guo et al.^9^ found that the crack initiation and penetration time lengthened with the increase in the number of dry–wet cycles, which indicated that the dry–wet cycles weakened the stiffness of the rock and enhanced its ductility. Mu et al.^10^ attributed the decline in rock stiffness primarily to the attenuation of the internal friction angle induced by dry–wet cycles. Additionally, following the energy dissipation theory, Chen et al.^11^ found that as the number of dry–wet cycles increased, the rock’s energy dissipation progressively rose. Drawing on this body of prior research, this paper conducts comprehensive physical and mechanical tests on red sandstone, parametrically delineating the effects of dry–wet cycles on degradation.

With the remarkable advancements in science and technology, coupled with the rapid evolution of rock mechanics testing methodologies, scholars have unveiled defects existing across various scales, ranging from micro to macro, within rock formations^12^. These defects could potentially contribute to the detrimental effects of dry–wet cycles. Through microscopic observation technology, researchers have found that widely distributed cracks inside rocks can provide an environment for the degradation of water^13–15^. Employing a variety of imaging technologies, such as scanning electron microscopy (SEM) as seen in studies by Coombes et al.^16^ and Chen et al.^17^, CT scanning experiments utilized by Fu et al.^18^ and Li et al.^19^, X-ray analysis employed by Yang et al.^20^, optical microscopy techniques demonstrated by Hou et al.^21^, and three-dimensional ray microscopy technology utilized by Xu et al.^22^, it was observed that the pore structure of rocks underwent varying degrees of alteration under the influence of drying and wetting cycles. This micro-level examination further elucidates the degradation effects of wet–dry cycles.

The above microscopic observation techniques are mainly used to describe the microscopic pore structure and composition of rocks under the action of dry–wet cycles. However, these studies have not directly established a link between the changes in pore structure and alterations in mechanical properties. In light of this, this paper initiates its investigation by elucidating the degradation of red sandstone under dry–wet cycles through a series of physical and mechanical experiments. Subsequently, leveraging microscopic observation technology, the paper offers a comprehensive description of the factors responsible for changes in pore structure and the resultant deterioration in strength. By establishing a direct connection between the microscopic damage characteristics and the macroscopic consequences on red sandstone, this research contributes essential insights, thereby offering a valuable reference point for the stability design of reservoir bank slopes.

## Sample preparation and test procedure

### Sample preparation

**Figure 1:**
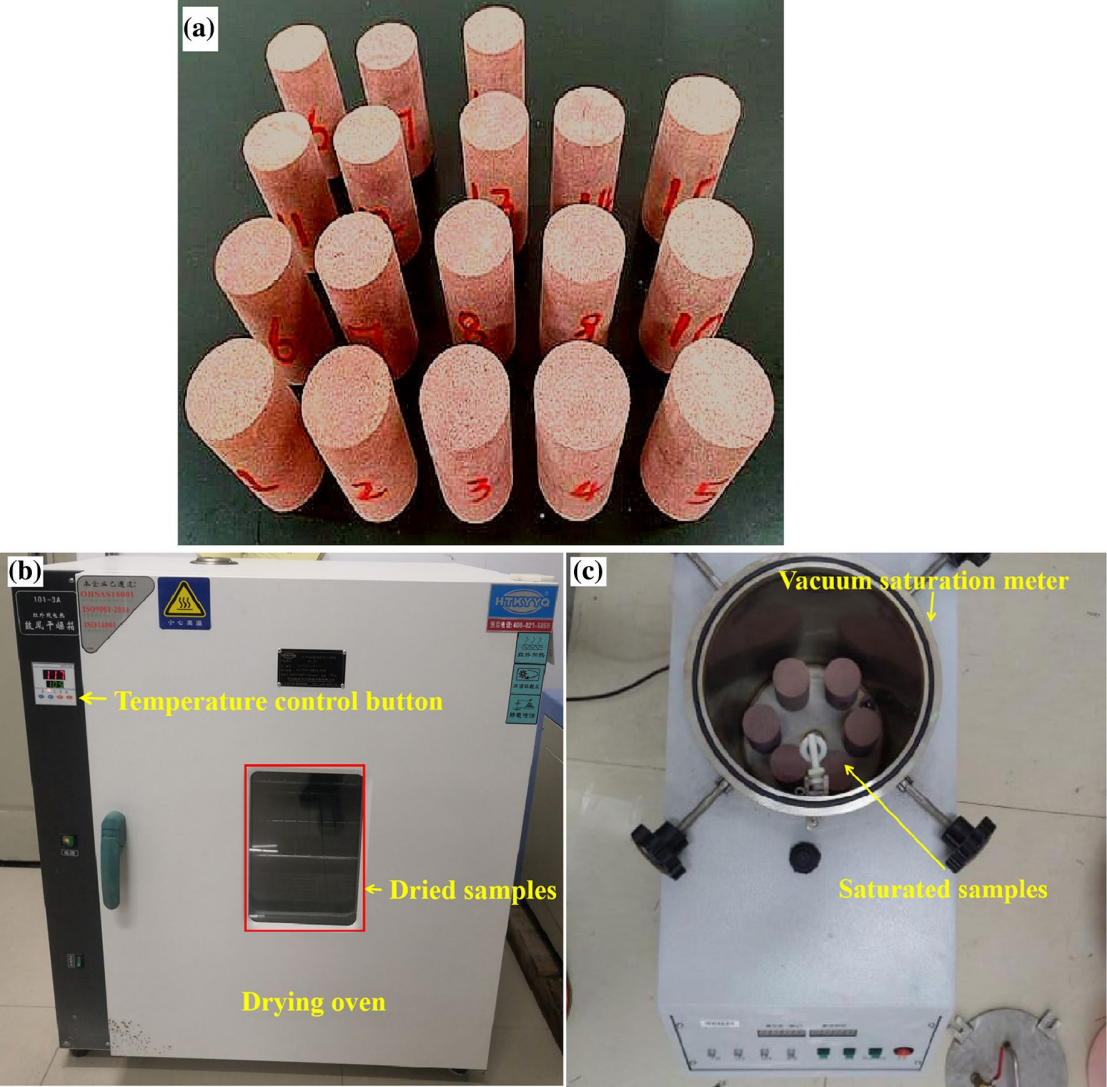
Dry–wet test apparatus: (**a**) sample preparation, (**b**) drying box and (**c**) vacuum saturation meter.

The samples were collected from the slope of the area of fluctuating water level in the Three Gorges Reservoir in Chongqing, China, and they were typical red sandstone. The sample had dense structure, the mineral particles that were are coarser than the matrix and the colour of red flesh, and density approximately 2380 kg/m^3^. A total of 25 test samples were processed, which were standard cylinders with a diameter of 50 mm and a height of 100 mm. The ends of the samples were ground and smoothed, and the flatness of the samples met the basic requirements of international rock mechanics tests, as shown in Fig. 1a.

### Test procedure

#### Physical test procedure

To better explore the effects of dry–wet cycles on the physical properties of red sandstone, five different dry–wet cycles were designed: 0, 10, 20, 30, and 40 cycles. The procedure for conducting the wetting–drying cycles can be elucidated as follows: (1) Initial dimensions and mass of the specimen were meticulously ascertained, including its height and diameter, and subsequently documented. (2) In alignment with the guidelines stipulated by Chinese Standard DZ/T 0276.1-2015, each sample was subjected to thermal treatment in an oven, calibrated to a constant temperature of 105 °C, for a duration of 12 h. After cooling to room temperature in a desiccator, the samples were then removed and weighed. (3) Thereafter, the specimens were saturated by being submerged in water under a vacuum pressure of 100 kPa for an additional 12-h timeframe. Upon reaching a state of saturation, they were extricated from the aqueous environment and any residual surface water was expeditiously eliminated. (4) Steps 1 through 3 were reiterated iteratively for successive cycles, continuing until the predetermined number of cycles was achieved. Subsequent to the completion of each cycle, the samples were once again subjected to weighing. Finally, dry–wet cycles were performed on the samples for the designed number of cycles (as shown in Fig. 1b,c). During a dry–wet cycle, the dry mass and saturated mass were recorded, and the porosity was calculated after each dry–wet cycle.

#### Micro-scale test procedure

**Figure 2:**
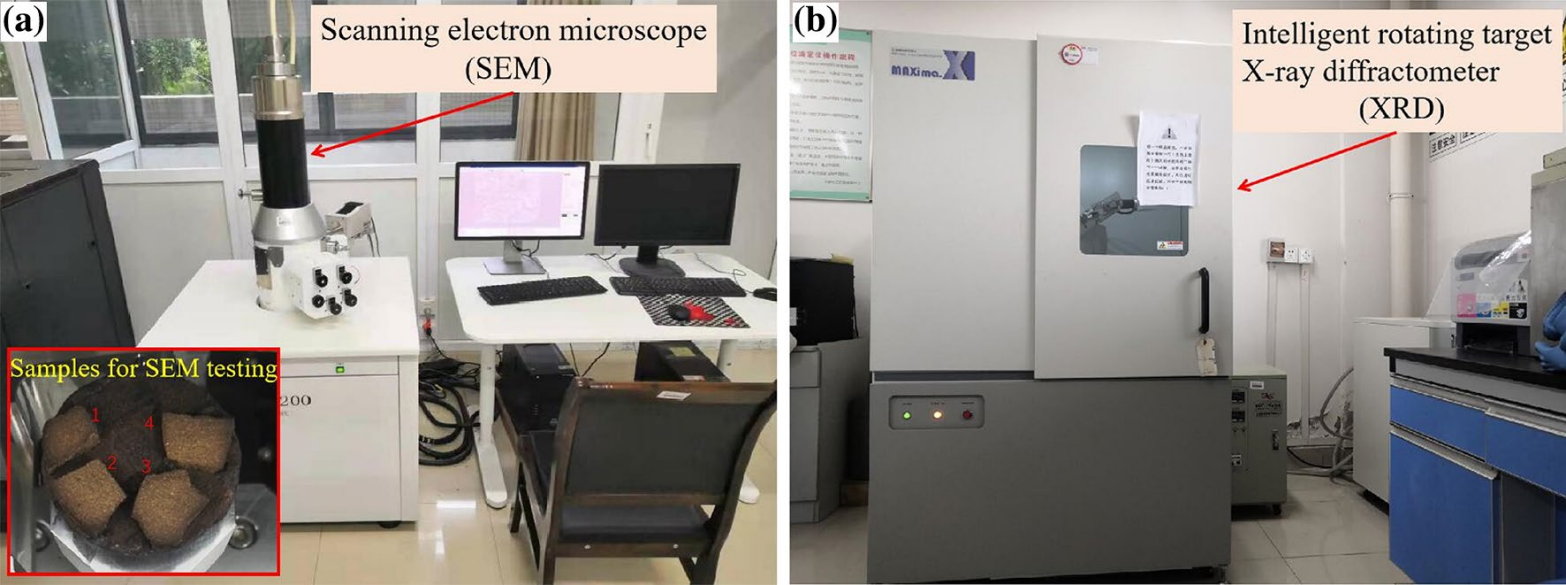
Microscopic test apparatus: (**a**) KYKY-EM6200 electron microscope scanner and (**b**) intelligent rotating target X-ray diffractometer.

To facilitate a comprehensive analysis of the evolving physical and chemical attributes throughout the dry–wet cycles, red sandstone block samples were meticulously prepared. These specimens were designed with an approximate surface area of 1 cm^2^ and a thickness ranging from 2 to 3 mm. Following their exposure to a series of dry–wet cycles, these samples were subsequently subjected to tests aimed at evaluating their mesoscopic properties. Scanning electron microscopy (as shown in Fig. 2a) was used to collect microscopic images of samples after different dry–wet cycles. Parts of samples after different cycles were ground into powders that were passed through an 80 μm sieve, and the changes in mineral components were analysed with an intelligent rotating target X-ray diffractometer (as shown in Fig. 2b).

#### Mechanical test procedure

**Figure 3:**
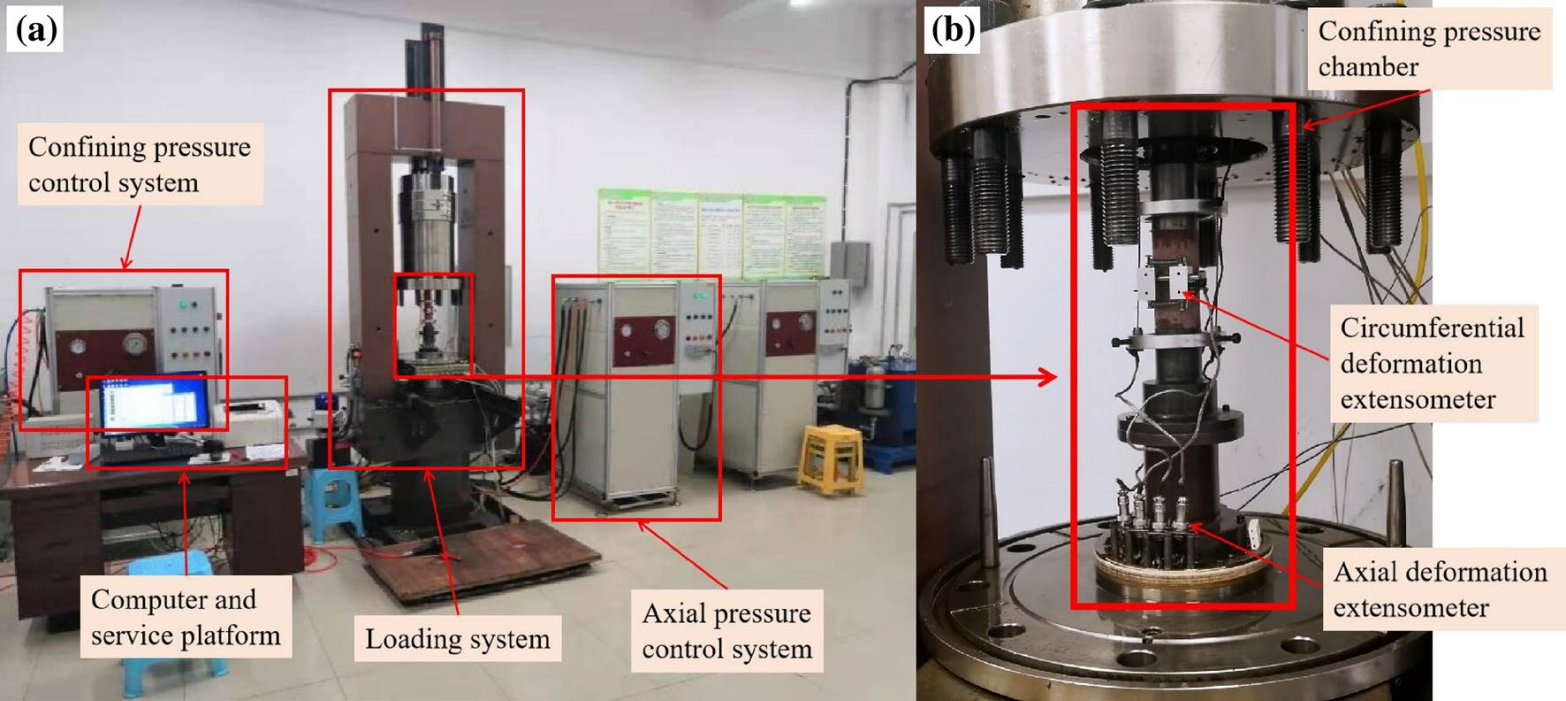
TFD-2000 microcomputer servo control rock triaxial rheological testing machine: (**a**) TFD triaxial testing machine and (**b**) loading system.

A TFD-2000 microcomputer servo-controlled rock triaxial testing machine (as shown in Fig. 3) was used for the triaxial compression test, the stress-controlled loading method was adopted, and the loading speed was 0.5 MPa/s until the specimen failed. To explore the influence of confining pressure, the confining pressure was designed to be 5, 10, 20, or 30 MPa.

## Test results and analysis

### Basic physical properties

#### Dry and saturated mass

**Figure 4:**
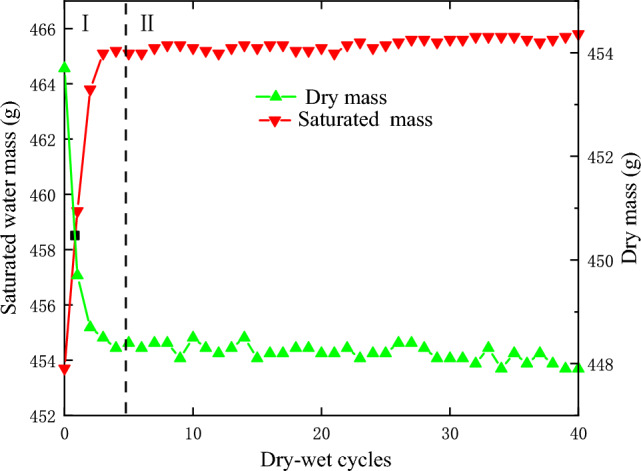
Mass change of red sandstone after different dry–wet cycles.

Figure 4 shows the graph of dry and saturated mass after different numbers of dry–wet cycles. The curves of the test mass and cycles in Fig. 4 could be divided into two stages from 5 cycles. In the first stage, the dry mass decreased significantly while the saturated mass increased significantly with increasing number of cycles, the slope of the curve was relatively large, and the mass varied greatly. In the second stage, the slopes of the two curves changed smoothly, and the dry mass decreased slightly while the saturated mass increased slowly. The curves showed that the rock was in a relatively steady state.

### Mechanical properties

#### Triaxial compression test results

**Figure 5:**
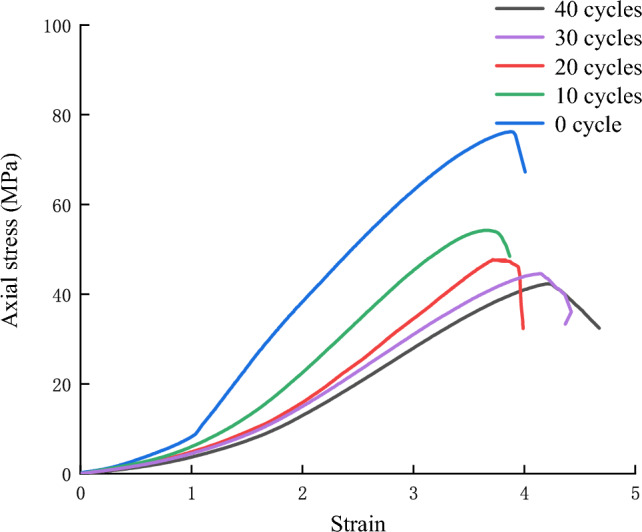
Uniaxial stress–strain curves of red sandstone with different cycles.

Figure 5 shows the stress‒strain curve of the red sandstone during the uniaxial compression test. With increasing number of dry–wet cycles, the compaction stage significantly shortened, and the slope of the linear elastic stage gradually decreased. Moreover, the peak strength dropped significantly from 0 to 10 cycles and then showed a slow decrease. Figure 5 also shows that the post-peak stage of the rock samples was in a brittle state after 20 dry–wet cycles, and when the cycles continued, the rock samples began to appear in a ductile state in the post-peak stage.

**Figure 6:**
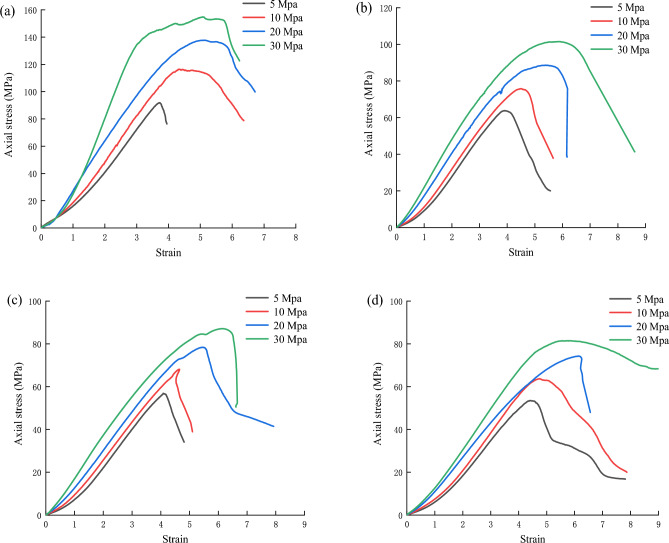
Triaxial stress–strain curves of red sandstone with different cycles: (**a**) 0 cycle, (**b**) 10 cycles, (**c**) 20 cycles, (**d**) 30 cycles.

Figure 6 shows the stress‒strain curves of the red sandstone under triaxial compression conditions after different dry–wet cycles. Rock samples underwent compaction, elasticity, plastic deformation and peak strength stages during different dry–wet cycles. Interestingly, after the same number of dry–wet cycles, the compaction stage was much shorter, and the slope of the linear elastic stage increased, while the peak strength stage obviously increased with increasing confining pressure. In addition, the compaction stage and plastic stage showed increasing trends, while the elastic stage and peak strength showed decreasing trends with increasing number of dry–wet cycles under the same confining pressure. In general, the results showed that the presence of confining pressure and different numbers of dry–wet cycles significantly affected the different stages.

**Figure 7:**
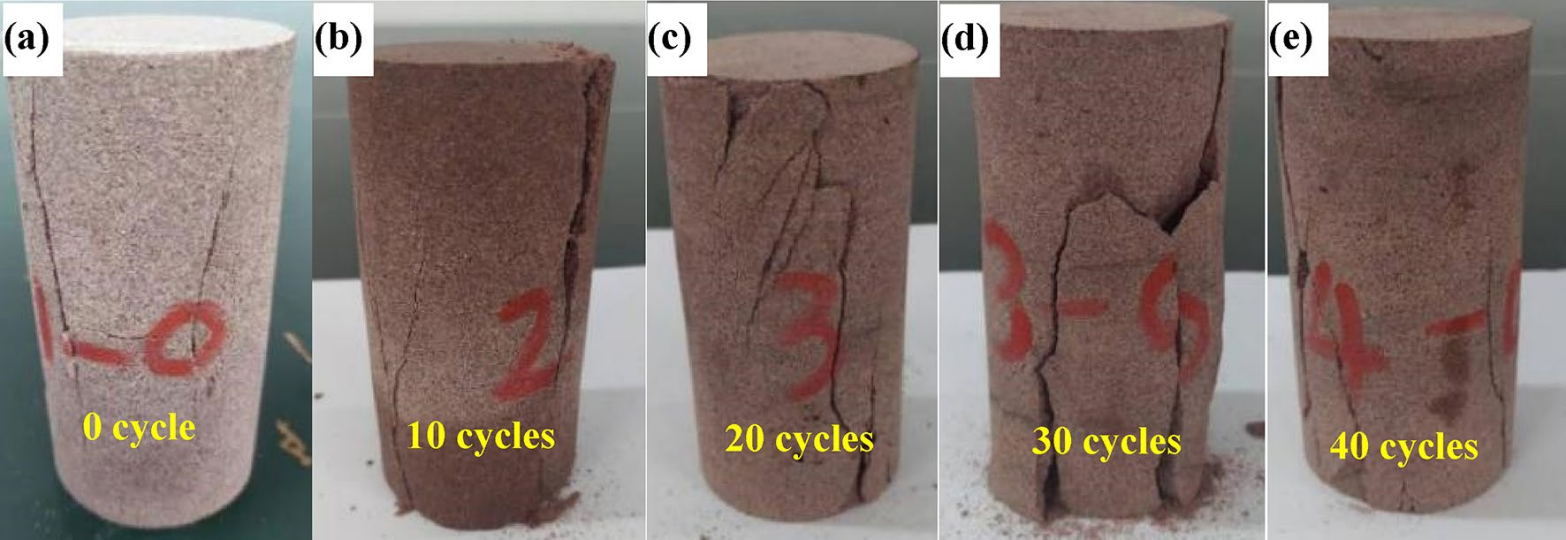
The failure modes of uniaxial tests with different cycles.

**Figure 8:**
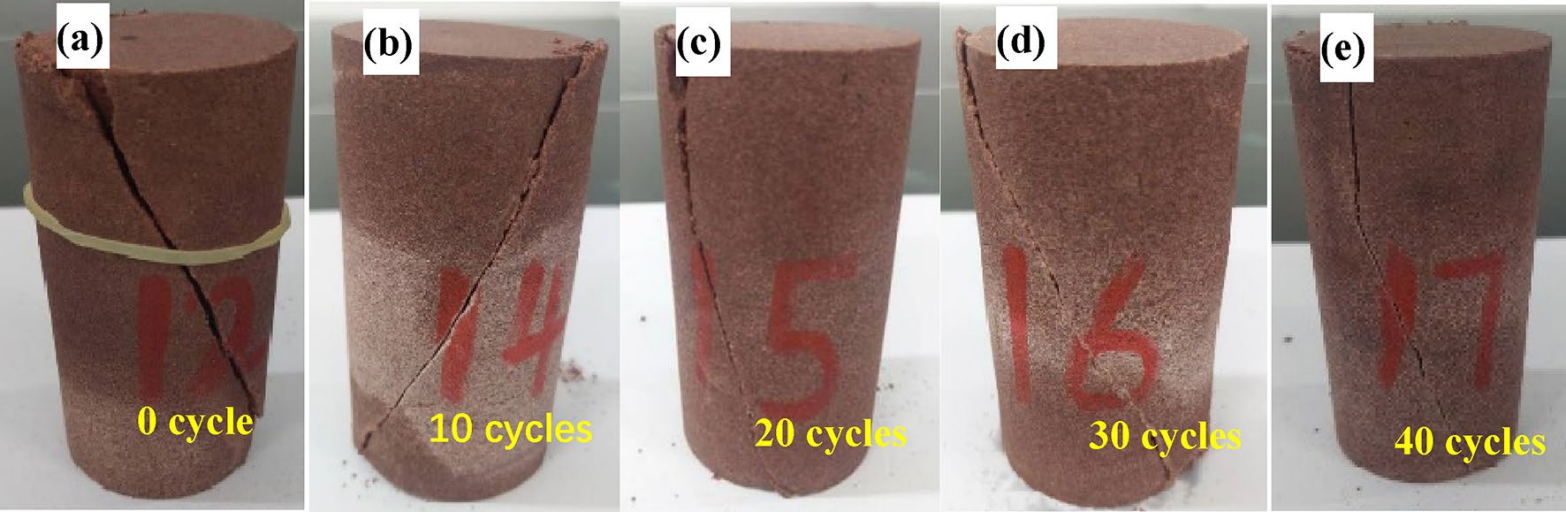
Triaxial test failure modes of different cycles under 20 Mpa.

Figures 7 and 8 shows the failure modes of red sandstone samples after different numbers of dry–wet cycles. During the formation of rocks, intricate geological processes invariably introduce internal defects such as pores and cracks as part of the diagenesis phase. The stochastic distribution of these defects, varying in both magnitude and orientation, significantly influences the rock’s failure modality. Specifically, the degradation of the internal microstructure of red sandstone under varying frequencies of dry–wet cycles results in distinct levels of rock weathering, which in turn, shape its macroscopic failure patterns. In its pristine state, red sandstone undergoing uniaxial compression failure exhibits discernible tensile failure, characterized by multiple fracture planes. Upon completion of 10 cycles, the failure mode continues to display prominent brittle tension, but an increasing incidence of damage becomes evident. Morphological analysis of the red sandstone subjected to 20 and 30 dry–wet cycles reveals that fractures increasingly intersect at oblique angles. Simultaneously, there is a notable increase in the frequency of cracks, accompanied by the emergence of localized fracture surfaces within the rock’s structure. Consequently, the fracture dynamics transition from isolated longitudinal cracks to a more intricate, composite pattern. This suggests that as the number of dry–wet cycles increases, the rock’s inherent brittleness begins to diminish, giving way to more ductile or plastic failure characteristics. At this juncture, the failure traits can be aptly categorized as a composite of tensile and shear failures. Upon reaching 40 dry–wet cycles, a conspicuous transformation occurs in the rock’s failure modality. The samples predominantly display shear failure, characterized by multiple shear planes. Among these, one dominant fracture surface, oriented at a 45-degree angle, is consistently observed. Additional, secondary fracture planes intersect this primary plane, underscoring the localized impact of dry–wet cycle-induced weathering on the rock sample. This pattern reveals a transition in the red sandstone from an initially brittle state toward increasing plasticity. Correspondingly, as the number of dry–wet cycles intensifies, the fractures not only elongate but also increasingly overlap, resulting in a more complex fracture surface topology.

### Peak strength

**Figure 9:**
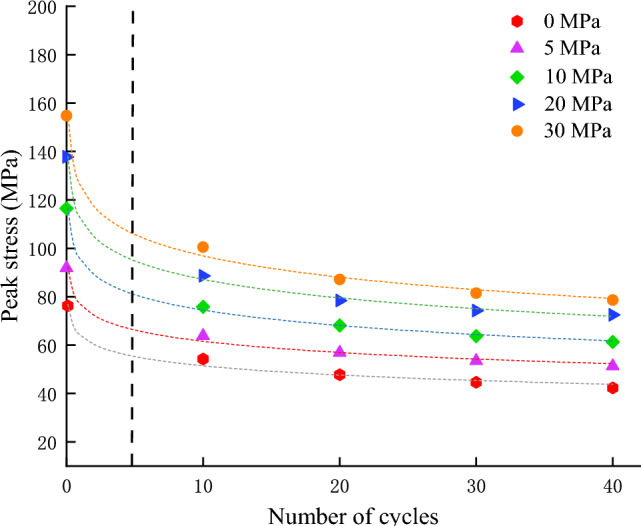
The relationship between the peak strength of red sandstone and the dry–wet cycles under different confining pressure levels.

**Figure 10:**
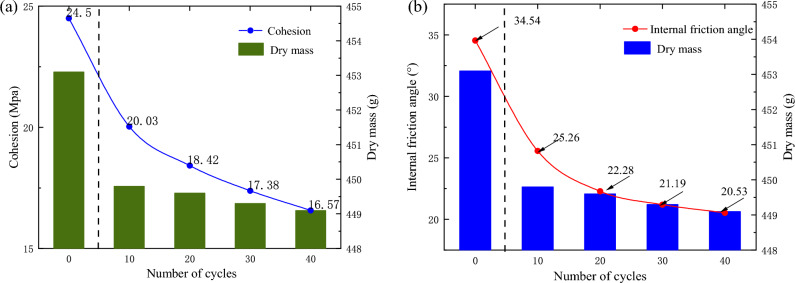
Variation of shear strength parameters: (**a**) variation of cohesion, (**b**) variation of internal friction angle.

Figure 9 shows the relationship between the peak strength of red sandstone under different confining pressure levels after different numbers of dry–wet cycles. The peak strength decayed exponentially with increasing number of cycles. The average peak strengths of the red sandstone samples after 0 cycles were 91.89, 116.44, 137.72, and 154.79 MPa when the confining pressures were 5, 10, 20 and 30 MPa, respectively. Under the same confining pressures, the average peak strengths of the red sandstone samples were 63.83, 75.83, 88.62, and 100.50 MPa after 10 dry–wet cycles, which decreased by 30.54%, 34.88%, 35.65%, and 35.07%, respectively. Compared to the 10 cycles, the average peak strengths of the red sandstone samples after 20 cycles were 56.88, 68.13, 78.39, and 87.10 MPa, respectively, which were reduced by 10.89%, 10.15%, 11.54%, and 13.33%, respectively. Compared to the 20 cycles, the average peak strengths of the red sandstone samples were 53.52, 63.72, 74.27, and 81.46 MPa, which were reduced by 5.9%, 6.47%, 5.26%, and 6.36%, respectively. Finally, the average peak strengths of the samples were 51.34, 61.27, 72.49, and 78.60 MPa, which decreased by 4.07%, 3.84%, 2.40%, and 3.51%, respectively, compared to 30 cycles. Thus, Fig. 10 showed that there was a significant difference in the peak strength of red sandstone from 0 to 10 cycles, and the peak strength decreases to a relatively certain value when the number of cycles increased.

#### Cohesion and internal friction angle

Figure 10 shows the relationship between the cycles and shear strength parameters under a confining pressure of 10 MPa. The cohesion decreased gradually with increasing cycles. The cohesion and the internal friction angle gradually decreased with increasing number of cycles, and there was a significant decrease from 0 to 10 cycles. Furthermore, the decrease was similar to the change in peak strength.

The changes in the cohesion and internal friction angle were consistent with that of the dry mass. This could be explained by the changes in the dry mass with the increase in number of dry–wet cycles, and consequently, the porosity varied. That is, under the condition of a constant volume, more cycles led to more pores and cracks and eventually a heavier saturated mass. In other words, the lighter the dry mass was, the more pores there were in the samples. In addition, the increase in porosity reduced the internal cementation of the sample and reduced the cohesion, the internal gradation of the sample became worse, and the internal friction angle decreased consequently.

### Mechanism of strength deterioration

#### Links between mineral composition and cycles

**Figure 11:**
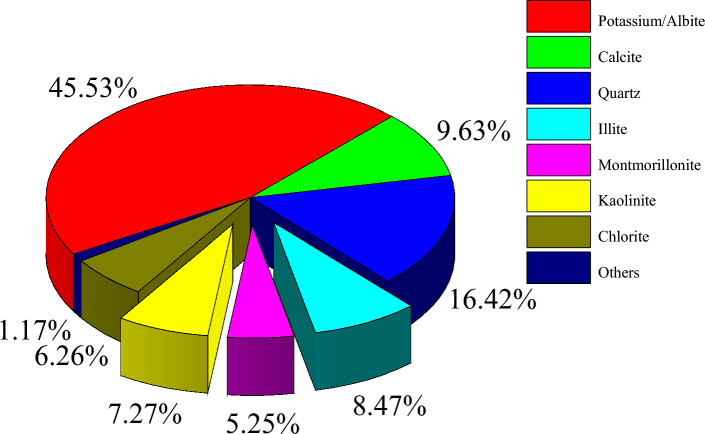
The original mineral composition content of the sample.

According to the X-ray diffraction test results, the main components of the detrital minerals in the original sample were potassium/albite ($\text{Na/KAl}{\text{Si}}_{3}{{\text{O}}}_{8}$), quartz (${\text{Si}}{\text{O}}_{2}$), calcite (${\text{CaC}}{\text{O}}_{3}$) and some soil minerals illite (${\text{K}}_{0.9}{{\text{A}}{\text{l}}}_{2.9}{\text{Si}}_{3.1}{\text{O}}_{10}{\left({\text{OH}}\right)}_{2}$), kaolin (${\text{K}}_{0.9}{{\text{A}}{\text{l}}}_{2.9}{\text{Si}}_{3.1}{\text{O}}_{10}{\left({\text{OH}}\right)}_{2}$), chlorite $({\text{Al}}_{4}{{\text{Si}}}_{4}{{\text{O}}}_{10}{\text{(OH)}}_{8}$), montmorillonite (${\text{(Na, Ca)}}_{0.33}{\text{(Al, Mg)}}_{2}\text{[}{\text{Si}}_{4}{{\text{O}}}_{10}\text{]}{\text{(OH)}}_{2}\cdot {\text{n}}{\text{H}}_{2}$ O), and small amounts of other minerals were mixed. Its original mineral composition content is shown in Fig. 11.

One of the most reliable bases for describing the crystallinity of minerals is the half-peak width. The crystallinity of each mineral is different, and the change in crystallinity represents the change of mineral particles. In this study, the mineral composition of red sandstone changed with the number of dry–wet cycles. As shown in Table 1.

**Table 1:** Half-peak width of each mineral composition.

Number of cycles	Quartz	Potassium/Albite	Calcite	Montmorillonite	Illite	Kaolin	Chlorite
0	0.241	0.262	0.255	0.366	0.351	0.367	0.296
10	0.177	0.388	0.339	0.381	0.186	0.202	0.186
20	0.196	0.401	0.202	0.204	0.162	0.177	0.255
30	0.204	0.229	0.132	0.236	0.202	0.267	0.234
40	0.213	0.109	0.245	0.234	0.267	0.296	0.245

**Figure 12:**
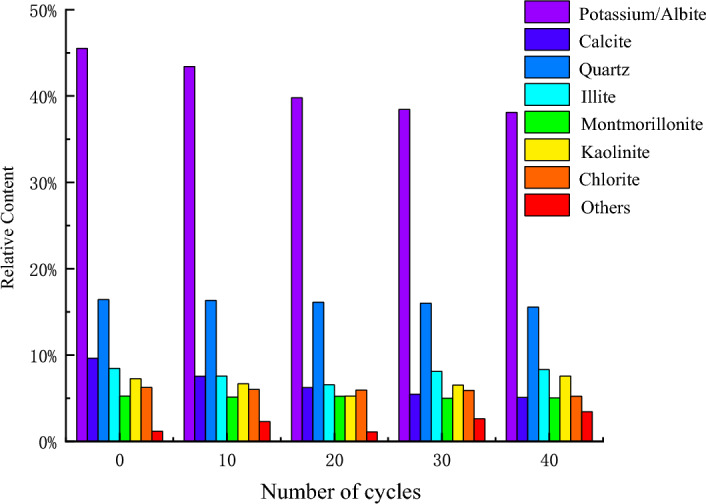
Contents of mineral particles after different dry–wet cycles.

Figure 12 shows the content of each mineral particle after different cycles. The contents of kaolinite and illite decreased initially and then slowly increased with the change in dry–wet cycles. Additionally, potassium/albite decreased, calcite decreased, while montmorillonite and chlorite showed a slightly decreasing trend. Because montmorillonite has a strong ability to undergo expansion, there was little change in montmorillonite content. Due to the dissolution and swelling properties of calcite, the content was reduced. Simultaneously, the dissolved and hydrolysed properties of potassium/albite reduced the content of these mineral. As water molecules infiltrated the interlayer particles, new illite and kaolinite formed after dissolution, and the contents of these two minerals tended to increase slightly. The relative contents of kaolinite, illite, potassium/albite, and calcite clearly changed after different dry–wet cycles.

### Links between microfeatures and cycles

**Figure 13:**
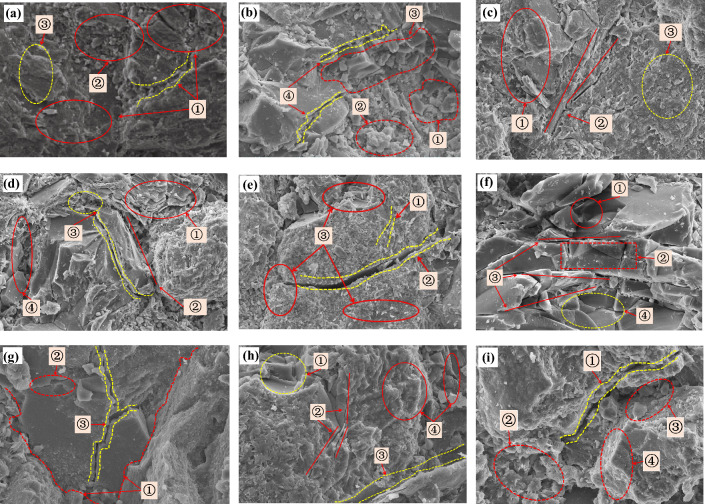
Relationship between micro-fracture locations and mineral grains after different dry–wet cycles. (**a**) ➀ Quartz crystal pores, ➁ Intergranular clay mineral filling, ➂ Clay aggregates. (**b**) ➀ Quartz particles, ➁ Illite/montmorillonite mixed layer, ➂Feldspar with cleavage planes, ➃ Original microcracks. (**c**) ➀ Kaolinite interlayer extrusion, ➁ Clay inter-connecting holes and seams, ➂ Small-scale primary pores. (**d**) ➀ Flaky kaolinite connections weaken, ➁ Crystal and filler connecting holes, ➂ Seam connected, ➃ Crystal edge pore extrusion. (**e**) ➀ Original seam, ➁ Unicom holes become longer, wider and deeper, ➂ Increased debris particles Decrease in roundness of crystal surface. (**f**) ➀ Dissolution pores, ➁ Transgranular fracture, ➂ Crack along the crystal plane, ➃ Flake illite, kaolinite extrusion. (**g**) ➀ Skeleton particles, ➁ Flake illite peels off to produce cracks, ➂ The primary throat is enlarged and connected. (**h**) ➀ Clay exfoliated holes, ➁ Connecting pores and fissures between quartz particles and clay minerals, ➂ Original throat enlargement, ➃ Clay expansion crack extrusion and filling. (**i**) ➀ Kaolinite interlayer extrusion, ➁ Kaolin, illite increased in pompom shape, ➂ Chlorite increased, ➃ Poor roundness, blurred edges and corners).

Figure 13 shows the SEM images of the relationship between positions of micro-scale fractures and mineral particles after different dry–wet cycles. The SEM images of the samples after different numbers of cycles were used to analyse the sandstone microstructure from the perspectives of mineral particle characteristics, the state of clay minerals, pore characteristics and crack growth. To have a clearer understanding of the changes in the microscopic characteristics during the whole dry–wet process, it was divided into three stages as follows:

1. *The first stage* at the initial stage of the dry–wet cycle (before 5 cycles). According to the SEM analysis of the representative rock samples (Fig. 13a–c), combined with the results of X-ray diffraction analysis, this red sandstone was mainly composed of quartz and feldspar particles interspersed with calcite particles. The intergranular pores and cracks were filled with kaolinite and illite as the main clay minerals. At this time, the pores were dominated by the holes and cracks between quartz particles, and the size was large, while the small pores between the particles were filled with clay particles. The internal pores of red sandstone were dominated by intergranular pores and contained many primary cracks. Specifically, the intergranular pores of clay minerals such as kaolinite, illite and montmorillonite were dense and small in size. In the initial state, the interstitial spaces between particles are densely packed with clay particles, rendering the sample in a relatively compact configuration. Following the first 5 cycles, the partially weathered clay particles within these pores undergo physical and chemical interactions with water, precipitating a marked decline in cohesion.
2. *The second stage* as the number of dry–wet cycles increased (from 5 to 10 cycles). The number of debris particles increased, and the infiltration of water molecules produced many new cracks and pores, and these cracks basically developed along the original cracks. As shown in Fig. 13d, the connection between the flaky kaolinites weakened, the volume was loose, the sizes of the pores between the clay particles increased, and their number also increased. As shown in Fig. 13e, the length, width and depth of the connected pores and fractures increased under the action of circulation, while some dissolved pores appeared. From Fig. 13f, the cementation mode and compactness near the bedding plane of flaky kaolinite and illite changed. The extrusion of clay minerals such as kaolinite and illite and between clay minerals and the quartz framework also increased. During this phase, the majority of the partially weathered clay particles have been depleted from the interstitial spaces. As water molecules infiltrate the material, new pores are generated and coalesce with the existing pore network to establish a newly stabilized structure under the compressive influence of the skeletal framework. Hence, the diminished cohesion at this juncture is primarily attributable to the loss of a fraction of clay particles and the emergence of additional pores. However, the rate of cohesion decrease decelerates at this stage, mainly due to two factors: first, a reduced loss of clay particles, and second, the integration of new pores with existing ones to form a newly stable structure.
3. *The third stage* with further immersion of water molecules (after 10 cycles). From Fig. 13g, due to the constraint of the quartz skeleton, the small pores were filled and squeezed, the clay minerals near some quartz particles were reduced, and most of the primary throats continued to extend and connect. As shown in Fig. 13h, the connection between clay particle aggregates was lost, and pores and cracks appeared within the clay minerals. As shown in Fig. 13i, the clay particles continued to expand, and the kaolinite interlayers were squeezed. Kaolin and a small amount of illite attached to the surface of the framework particles, partially filled in the pores or cracks and were removed by the pore fluid. Then, the amount of pompom-shaped fine-grained illite and kaolinite increased and adhered to the surface of skeleton particles, and the number of flaky and laminated kaolinite decreased rapidly. By comparison, mineral aggregates have low identification, poor roundness, and volume expansion at the same location. At this later stage, the interstitial clay particles are essentially exhausted. Both new and pre-existing pores progressively coalesce into connected fractures under the influence of water molecules. This phenomenon elucidates the heightened severity of sample rupture following the dry–wet cycles. The newly formed interconnected cracks establish a relatively stable internal architecture under the gravitational pull exerted by the sample’s own skeletal framework. Consequently, the pace of cohesion reduction experiences further deceleration at this point.

## Discussion on the microscopic mechanism of strength deterioration

### Water film expansion stress

From the perspective of mineral composition, the chemical reactions of minerals promote the deterioration of red sandstone through argillization, hydrolysis and ion exchange. Under natural conditions, the reaction proceeds spontaneously when water seeps into the red sandstone cracks. Specifically, the hydration of calcite into gypsum increases by 1.5 times, and the hydration of illite is the main reason for the expansion. The reaction equations are expressed as follows^23,24^.

The chemical reaction process of dissolution expansion of calcite:

1 $${\text{CaC}}{\text{O}}_{3}+ \text{C} {\text{O}}_{2}\text{+}{\text{H}}_{2}{\text{O}}\to {\text{Ca}}^{2+}+ \text{2HC} {\text{O}}_{3}^{-}.$$

The hydration swelling chemical reaction process of illite:

2 $${\text{K}}_{0.9}{\text{Al}}_{2.9}{\text{Si}}_{3.1}{\text{O}}_{10}{\text{(OH)}}_{2}+{\text{n}}{\text{H}}_{2}{\text{O}}\to {\text{K}}_{0.9}{\text{Al}}_{2.9}{\text{Si}}_{3.1}{\text{O}}_{10}{\text{(OH)}}_{2}\cdot {\text{n}}{\text{H}}_{2}{\text{O}}.$$

**Figure 14:**
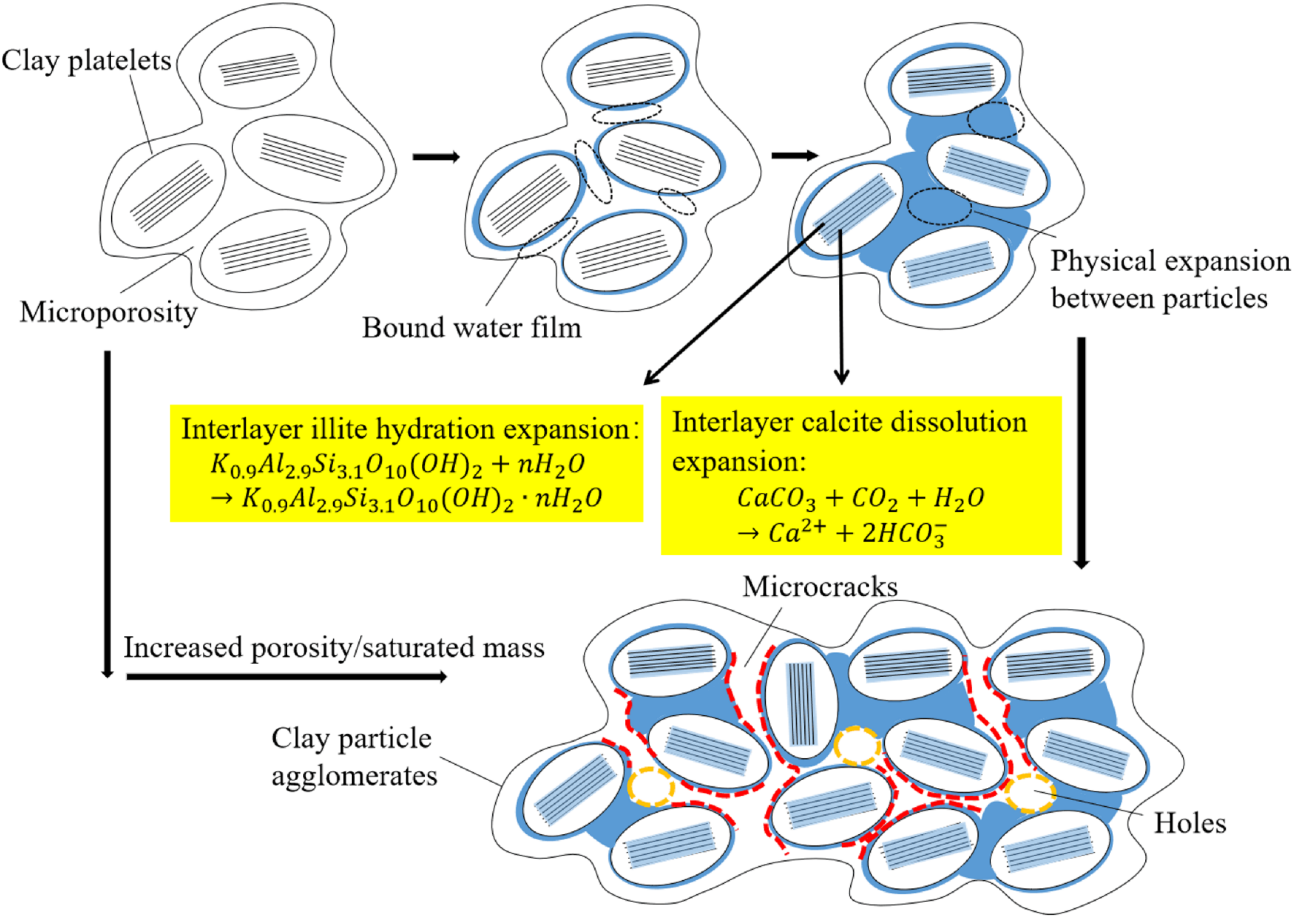
Water swelling and deterioration.

Figure 14 shows the processes of water absorption and expansion. There were many micropores in the primary red sandstone. Clay minerals are generally in the form of aggregates. The sandstone continued to absorb water and the water film thickened, and the clay aggregates became larger. In the case where there was little change in the total volume, the aggregates squeezed one other to produce an expansion effect. In addition, the expansion stress made the internal cohesion force of the red sandstone smaller, and the structure was loose. Finally, microcracks and pores formed. The porosity increased after the mineral volume expanded, which also led to an increase in the saturated mass. That is, as the porosity increased, the particle gradation deteriorated, and then the internal friction angle decreased.

### Extension of microcracks

**Figure 15:**
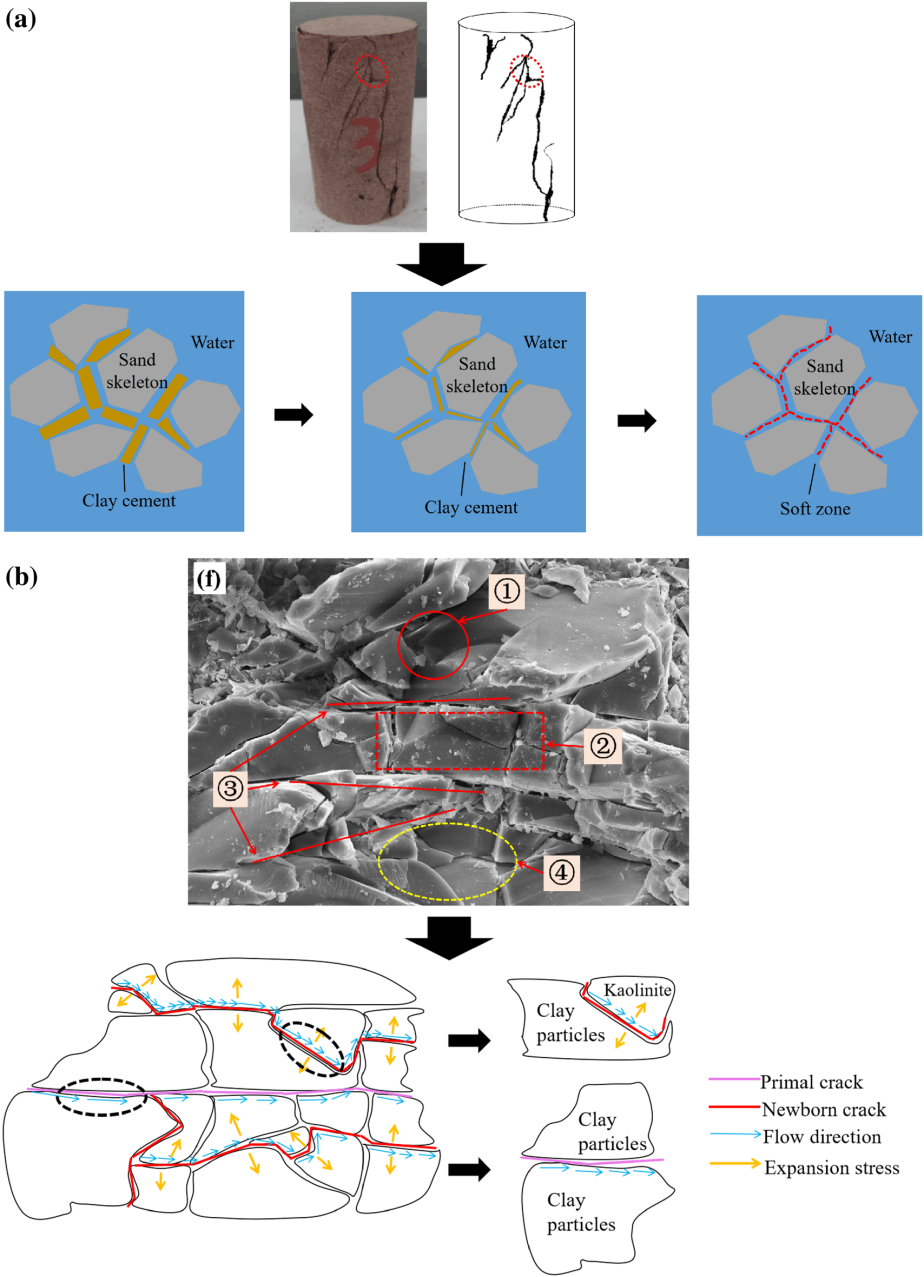
Local microscopic fracture and extend: (**a**) The soft zone formed by the local clay accumulation site and water interaction, (**b**) development of local microscopic fractures.

In Fig. 15a, the failure mode (Fig. 7c) was selected, locally enlarged and analysed. Many microcracks were present in the areas with high concentrations of clay particles because of immersion, adsorption of water molecules and penetration of water flow, which produced conditions for the chemical reaction of water and formation of soft zones in the sandstone.

Figure 15b shows an SEM image of typical microscopic cracks (Fig. 13f). Once a microfracture formed, it grew. Then countless microscopic fractures connected and formed a dense band where multiple microscopic fractures connected and extended to macroscopic fractures. When water further infiltrated, the rocks distributed along the fault or fault zone further fractured and fine-grained X, forming highly fragmented rock fragments. Under the action of repeated dry–wet cycles, rock fragments repeatedly broke, the particle size continuously decreased, and the particles underwent relative friction sliding and rigid body rotation; thus, they could withstand large deformations and relative motions. This process is called fracture flow; it leads to the formation and growth of microscopic fractures and eventually decreases make the peak strength.

### Clay cement weakening

As shown in Fig. 13i, images obtained with a scanning electron microscope show that fluffy spheres of kaolinite and illite filled and attached to the surface of the skeleton, mainly because potassium feldspar reacted with partially electrolyzed ions in water during this process. The reaction (Eqs. 3, 4) newly formed illite and kaolinite, which destroyed the original particle skeleton and degraded the strength of the sandstone. The chemical reaction process of potassium feldspar dissolving into illite and kaolinite is as follows^24^.

3 $${\text{3KAl}}{\text{Si}}_{3}{{\text{O}}}_{8}+ \text{2} {\text{H}}^{+}+ \text{12} {\text{H}}_{2}{\text{O}}\to {\text{K}}{\text{Al}}_{3}{{\text{Si}}}_{3}{{\text{O}}}_{10}{\text{(OH)}}_{2}+{6}{\text{H}}_{4}{{\text{SiO}}}_{4}+{2}{\text{K}}^{+},$$

4 $${\text{2KAl}}{\text{Si}}_{3}{{\text{O}}}_{8}+{2}{\text{H}}^{+}+{9}{\text{H}}_{2}{\text{O}}\to {\text{Al}}_{2}{{\text{Si}}}_{2}{{\text{O}}}_{5}{\text{(OH)}}_{4}+{4}{\text{H}}_{4}{\text{Si}}{\text{O}}_{4}+2{\text{K}}^{+}.$$

**Figure 16:**
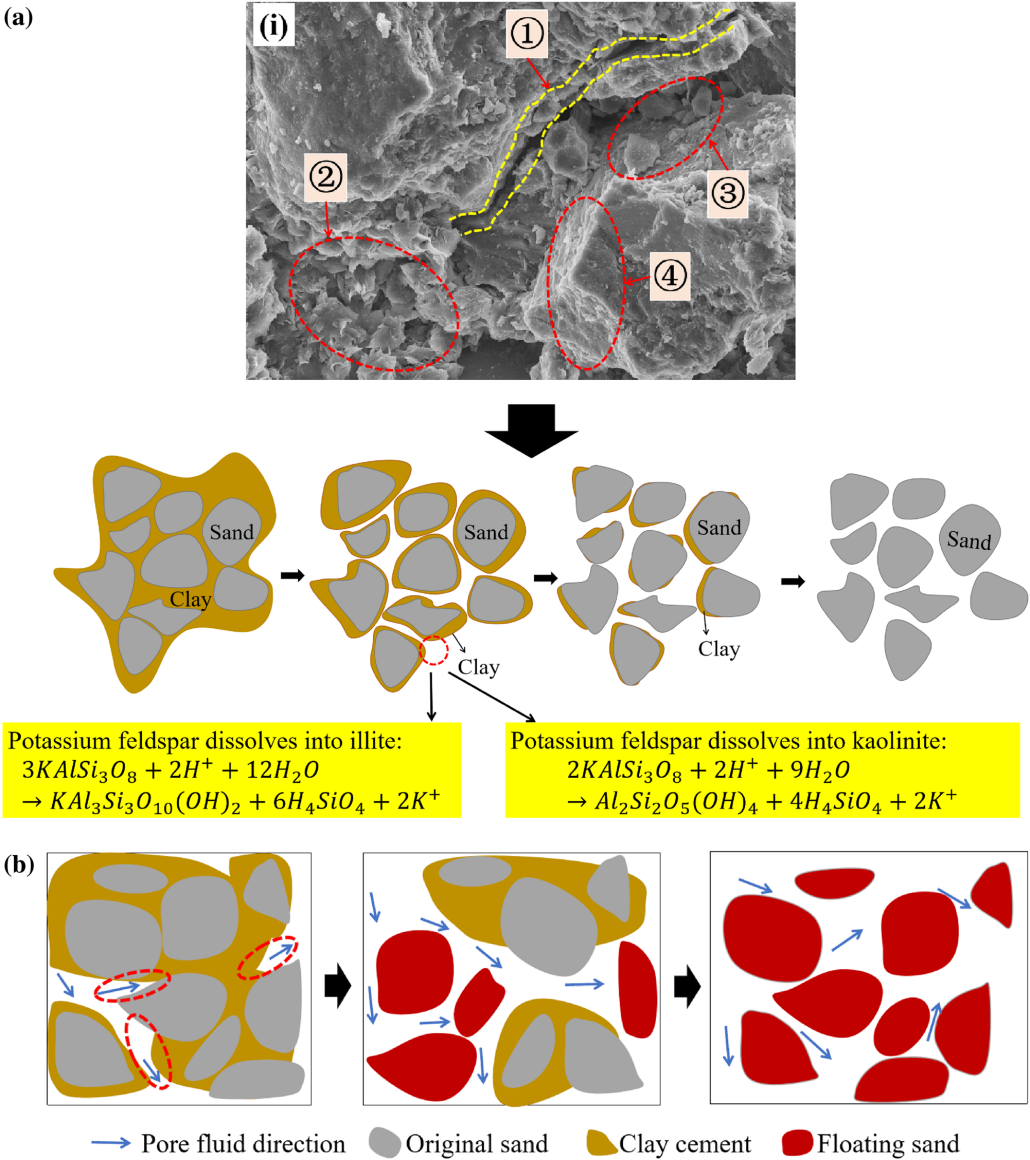
Clay mineral cementation weakening: (**a**) Local process of erosion and diffusion of clay, (**b**) local process of pore fluid action.

Figure 16a shows that because of the local microchemical reactions and effects of the pore fluid, the clay cement between particles gradually dissolved. Under multiple cycles of dissolution, the above chemical reaction became an irreversible process. From the perspective of the macroscopic level, the dry mass decreased, and on the microscopic level, the cementation became weaker and the internal structure was gradually destroyed. That is, the cohesion decreased as the clay cement decreased. In general, the microscopic changes in red sandstone were consistent with the trends in the physical parameters and shear strength parameters, which proved that the results obtained in this study were reasonable.

Figure 16b shows two pieces of evidence of the influence of pore fluid on rock mechanical strength. First, when the rock was rich in fluid, the rock strength could be reduced. Second, the fluid in the pores applied pressure, which is the pore pressure. When there is abnormal pore pressure in the rock, the effect is similar to that of reducing the confining pressure, which reduces the strength at which brittle failure occurs. When the pore pressure is almost equal to the confining pressure, the rock has a “floating effect”.

## Conclusions

In this paper, triaxial compression tests, basic physical tests and microscopic observations after different numbers of dry–wet cycles were used to study the loss of strength and mechanism for the degradation of red sandstone on the bank slope of the Three Gorges Reservoir, and the following conclusions were drawn:

1. Under the action of dry–wet cycles, the porosity and saturated mass of the red sandstone increased to a certain extent. Moreover, during the first 5 cycles, the changes in mass and porosity were most significant and increased steadily with increasing number of cycles.
2. The triaxial compression tests showed that the most significant effect on the loss of strength of red sandstone occurred from 0 to 10 cycles. The peak strength, cohesion and internal friction angle decreased the most obviously, and the development tended to stabilize, when the number of cycles increased. The variation in the shear strength parameters was similar to that of the physical properties.
3. With the increase in the number of cycles, the expansion effect of the water film and mineral particles led to an increase in the number of pores and cracks, and consequently, the porosity and saturated mass increased. Then, the gradation of the particles became worse and the internal friction angle decreased. Due to the effect of fracture flow and pore fluid, the microscopic cracks continued to extend, the cracks at the same positions became wider, the number of induced microcracks increased, and the peak strength decreased.
4. The changes in the half-peak width of red sandstone mineral particles and the content of mineral components indicated changes in the crystallinity of internal minerals. In addition, a series of hydrochemical reactions and physical effects weakened the clay cementation and reduced the content. The reaction of local mineral particles with water changed the relative content of minerals in the red sandstone, which led to a reduction in dry mass. In addition, the clay bond became weaker, resulting in less cohesion.

## Data Availability

Some or all data, models, or code that support the findings of this study are available from the corresponding author upon reasonable request.
